# Analysis of Flavonoids Bioactivity for Cholestatic Liver Disease: Systematic Literature Search and Experimental Approaches

**DOI:** 10.3390/biom9030102

**Published:** 2019-03-14

**Authors:** Juan Carlos Sánchez-Salgado, Samuel Estrada-Soto, Sara García-Jiménez, Sergio Montes, Jaime Gómez-Zamudio, Rafael Villalobos-Molina

**Affiliations:** 1 Instituto de Medicina Molecular y Ciencias Avanzadas, Mexico City 01900, Mexico; 2 Facultad de Farmacia, Universidad Autónoma del Estado de Morelos, Cuernavaca, MOR 62209, Mexico; saragarcia@uaem.mx; 3 Instituto Nacional de Neurología y Neurocirugía, Mexico City 14269, Mexico; montesergio@yahoo.com; 4 Unidad de Investigación Médica en Bioquímica, Hospital de Especialidades, Centro Médico Nacional Siglo XXI, IMSS, México City 06720, Mexico; jaime_gomez_zamudio@hotmail.com; 5 Unidad de Biomedicina, Facultad de Estudios Superiores-Iztacala, Universidad Nacional Autónoma de México, Tlalnepantla 54090, Mexico; villalobos@campus.iztacala.unam.mx; 6 Departamento de Bioquímica, Facultad de Medicina, Universidad Nacional Autónoma de México, Ciudad de México 04510, México

**Keywords:** flavonoids, systematic review, cholestasis

## Abstract

Flavonoids are naturally occurring compounds that show health benefits on the liver. However, there is little investigation about identification and evaluation of new flavonoid-containing drugs for cholestatic liver disease, one of the most common liver illnesses. We aimed to a systematic search regarding efficacy of flavonoids for treatment of cholestatic liver disease, and then evaluate naringenin (NG) as representative flavonoid in an obstructive cholestasis model. We searched for information of experimental and clinical studies in four major databases without time and language limits. Intervention was defined as any flavonoid derivate compared with other flavonoid, placebo, or without comparator. In addition, we evaluated NG on a bile duct-ligated model in order to contribute evidence of its actions. Eleven experimental reports that support the efficacy of flavonoids in cholestatic liver disease were identified. However, there was no homogeneity in efficacy endpoints evaluated and methodology. On the other hand, NG showed beneficial effects by improving specific metabolic (cholesterol and lipoproteins) and liver damage (bilirubin and alkaline phosphatase) biomarkers. The review lacks homogeneous evidence about efficacy of flavonoids in experimental settings, and is susceptible to risk for bias. NG only showed improvements in specific disease biomarkers. More investigation is still needed to determine its potential for drug development.

## 1. Introduction

Liver disease is a prominent health problem with an important epidemiological and economic burden worldwide, accounting for 25% of prevalence [1,2]. However, this estimation is inconsistent since there is no reliable and applicable diagnostic test yet [2].

Cholestasis is an impaired bile flow from liver to duodenum that triggers unspecific cellular damage, which initiates inflammatory and fibro-genic processes in the liver, as well as cirrhosis and hepatocellular cancer in advanced stages [3]. This alteration might be provoked by an impaired export of bile acids to bile canaliculi (intrahepatic cholestasis), or by physical obstruction of bile flow due to gall stones, parasites, or biliary tumor growth (extrahepatic cholestasis) [4].

The most accepted and well established experimental model that reproduces pathobiochemical alterations is the bile duct-ligated (BDL) rodent model [3,5]. The surgical bile duct ligation induces an obstructive cholestatic damage, which results in typical phenotype as in human cholestasis [3,5,6]. In this context, there are exhaustive efforts to found new therapeutic agents able to decrease pathological severity and progression of cholestatic liver diseases. Despite this, there are limited approved drug interventions for the treatment of this disease, i.e., ursodeoxycholic acid (UDCA) is the only approved oral drug for cholestatic liver diseases, such as primary biliary cirrhosis and obstructive cholestasis [7,8]. Additionally, silymarin, a mixture of flavolignans from Silybum marianum (milk thistle), has been proposed as an alternative for primary biliary cirrhosis due to its antioxidant and hepatoprotective properties [9,10,11].

Systematic reviews and meta-analysis have become increasingly important in decision making for drug discovery. In fact, this technique is a powerful evidence-based strategy to ensure there is justification for further research on bioactive molecules or re-planning research objectives [12]. A little investigation has been carried out on flavonoid efficacy in disease. Only a systematic review summarizes biological activities of prenylated flavonoids and suggests the structural implications for their bioactivities [13]. However, there is no information about efficacy of these compounds on cholestatic liver disease despite previous evidence for silymarin.

Previously we reported the beneficial effects of flavonoid-rich extract of *Cochlospermum vitifolium* bark for improvement of liver enzymes activity in BDL rat [14]. Then, HPLC analysis showed that one of its main bioactive compounds was naringenin (NG), a widely spread flavonoid in nature [15]. Recently, a chemical analysis revealed the presence of other flavonoids in the bark extract of this species [16].

This work summarizes and compares the efficacy endpoints reported for flavonoids in cholestatic liver disease, taking into account clinical and experimental settings, by systematic literature search in four major databases. Moreover, we evaluated the efficacy of NG in a BDL Wistar rat model in order to compare retrieved information from literature. To the best of our knowledge, this is the first systematic search of flavonoids as candidates for cholestasis therapy, as well as to show the biological effects of NG on the BDL rat.

## 2. Materials and Methods

### 2.1. Systematic Review

#### 2.1.1. Sources

We conducted a systematic search for peer-reviewed articles in four major databases: Pubmed (Medline), Cochrane Library, Trip Database, and Lilacs. PRISMA statement was followed in order to establish the evidence-based minimum set of items for reporting on systematic reviews.

#### 2.1.2. Eligibility Criteria

A PICO strategy was developed as described in Table S1. Briefly, the databases were searched using combinations of the following terms: flavonoid, cholestasis, and bile duct ligated. A species filter for human or animal was applied to identify clinical trials or experimental assays, respectively. Simple literature reviews, case reports, editorial letters, comments, as well as duplicated or no abstract articles were excluded. The searching did not consider time and language limits.

#### 2.1.3. Studies Selection

The inclusion criteria for the selection of manuscripts were: all articles containing keywords in title, abstract or full-text, as well as those reporting clinical trials or experimental studies of isolated flavonoids for the treatment of cholestatic liver diseases (primary biliary cirrhosis, primary sclerosing cholangitis, biliary atresia, or Alagille syndrome). Only with BDL were selected in order to compare our experimental data. The selection was conducted by two independent reviewers, who analyzed the articles for discrepancies.

#### 2.1.4. Meta-Analysis

A meta-analysis of the retrieved data could not be carried out due to methodological heterogeneity such as dosing, mode of administration, animal species, and time of exposure.

#### 2.1.5. Risk of Bias Assessment

In order to assess the risk of biases of the animal studies reported in this systematic review, we applied a modified version of Cochrane’s RoB tool denominated Systematic Review Centre for Laboratory animal Experimentation (SYRCLE). This tool considers six types of biases: Selection, performance, detection, attrition, reporting, and other biases, comprised of 10 items [17].

### 2.2. Experimental Studies

#### 2.2.1. Reagents and Materials

Naringenin ~95% were purchased from Sigma-Aldrich Co. (St. Louis, MO, USA). Other reagents and surgical supplies were purchased from local sources.

#### 2.2.2. Sample Preparation

Briefly, test samples were dissolved using a 10% aqueous solution of dimethylsulfoxide (DMSO). Dose of NG used was 50 mg/kg/day; the control group received 2 mL of vehicle.

#### 2.2.3. Animal Model

Male Wistar rats (250–300 g, b.w.) were used under laboratory conditions with standard rodent diet and water ad libitum. All of the protocols were conducted as established by Federal Regulations for Animal Experimentation and Care (NOM-062-ZOO-1999, SAGARPA) and approved by Scientific and Bioethics Committee of Facultad de Farmacia (UAEM).

All animals were anesthetized by intraperitoneal injection of sodium pentobarbital (35 mg/kg), shaved and sanitized using a surgical disinfecting solution. A 1 cm incision below xiphoid process was made to extract gastrohepatic epiplon and locate duodenum segment. Once bile duct was cleaned for connective tissue, a triple ligation was made on bile duct using non-absorbable monofilament nylon suture (Farmacéutica Internacional S.A. de C.V., Mexico City, Mexico), and cut in two segments. Unconscious animals were gently sutured and allocated in warmed room until recovery. Sham animals (surgery control group) were subjected to the same protocol, but without ligation.

After 24 h post-surgery, animals were randomly allocated in three experimental groups (*n* = 10 animals per group) and began to receive the treatment. The experimental groups were: Sham + vehicle, BDL + vehicle, and BDL + NG. Aseptic cleaning was made along experiments using povidone-iodine 10% solution as palliative care. After 10 days of treatment all animals were sacrificed by dislocation and blood samples extracted by cardiac puncture. ARRIVE guidelines were followed for reporting this assay.

#### 2.2.4. Biomarkers Quantification

Fresh collected blood samples were centrifuged at 3000 g for 10 min. Serum was separated from cell fraction and stored at −20 °C until analysis. Non-specific cholestasis biomarkers alanine transaminase (ALT), aspartate transaminase (AST), alkaline phosphatase (AP), and γ-glutamyltranspeptidase (GGT) activities) were determined as described by Rivera-Macías et al. [18].

Additionally, metabolic biomarkers were determined such as glucose, total cholesterol, triglycerides, very low-density lipoprotein (VLDL), high-density lipoprotein (HDL), and low-density lipoprotein (LDL). Quantitative enzymatic spectrophotometry was carried out using commercial kits (Wiener Labs, Mexico City, Mexico).

#### 2.2.5. Data Analysis and Statistics

Data shown are expressed as mean ± standard error of the mean (SEM) obtained from three independent experiments. Plots were constructed with Microcal™ Origin 6.0 software (Microcal Software Inc., Northampton, MA, USA). Statistical analysis was done with IBM^®^ SPSS^®^ software (IBM Corporation, Somer, NY, USA) by Student’s t-test and One-way ANOVA. Statistical significance was established when *p* < 0.05. Bonferroni post-hoc analysis was used for ANOVA.

## 3. Results

### 3.1. Systematic Review

**Figure 1:**
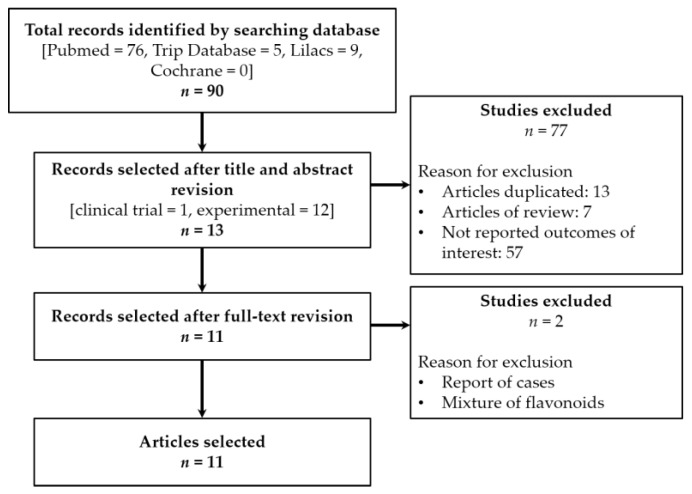
Flowchart showing the selection process for included studies on this systematic review.

Of all ninety articles identified in the primary search (Pubmed = 76, Trip Database = 5, Lilacs = 9, Cochrane Library = 0), we selected 12 of the experimental studies and one clinical trial (a pilot study on primary biliary cirrhosis). After full-text revision, we selected 11 articles for data discussion (Figure 1). All of them were on experimental studies on BDL rodents, as described in Table 1. Despite use of synonyms terms to retrieve a larger number of articles, we did not have more available information. Meta-analysis was not possible because of different animal species, route of administration, time of exposure, or methodology.

**Figure 2:**
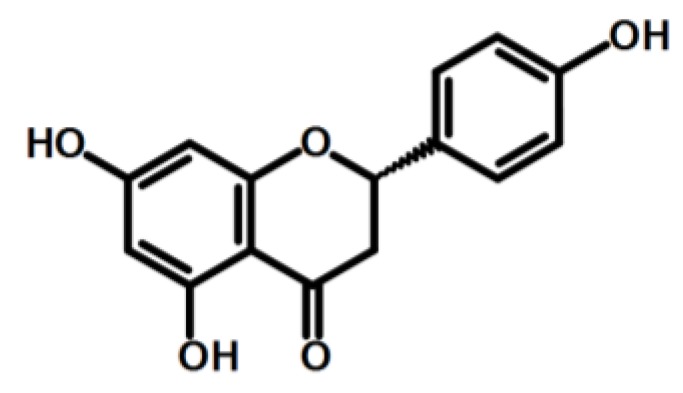
Chemical structure of naringenin (NG).

The major flavonoids identified from this search were diosmin, bacailin, quercetin, epigallocatechin 3-gallate, silybin or silibinin (major active component of silymarin), rutin (quercetin-3-O-rutinoside), and genistein (Table 1). Interestingly, no evidence was found about NG, one of the most naturally occurring flavonoid studied due of its health benefits on metabolic and cardiovascular diseases (Figure 2). This no evidence scenario encouraged us to evaluate NG as candidate for drug discovery, and contribute to experimental pharmacological data of flavonoids.

The biological processes altered on obstructive cholestasis are liver function, extracellular matrix components processing, redox status, and inflammation balance [3]. In this context, only diosmin, quercetin and rutin were evaluated for all these components (Table 1). Lin et al. showed that quercetin supplementation produced alleviation of liver histological integrity, serum biomarkers stabilization, reduction of fibrotic and inflammatory processes, and resolution of oxidative stress probably by modulation of bile duct proliferation and ductal reaction, blockade of mitogenic and fibrogenic signaling, and antioxidant effect [19]. Additionally, they evaluated rutin (quercetin-3-O-rutinoside), the O-glycoside compound that combine quercetin and disaccharide rutinose, on the same model and evaluated the same endpoints. The evidence showed that rutin had similar effects taking into account that it is converted to quercetin during metabolism [20].

On the other hand, Ali et al. reported data on efficacy of diosmin alone or in combination with sildenafil (erectile dysfunction approved drug with antioxidant and anti-inflammatory properties [21,22]) on the same model. Interestingly, diosmin alone was able to restore redox homeostasis by regulation of antioxidant enzymes GSR and SOD, a putative mechanism proposed for this molecule [23].

In order to compare parameters of the liver function and metabolic status on BDL-treated animals, we show tables of percentage change of each parameter vs. untreated cholestatic rats (Table 2 and Table 3). It should be noticed that only one study reported mortality as valid endpoint for evaluation of experimental efficacy [23]. Additionally, ductal enzymes AP and GGT were missing on several studies despite these biomarkers are pivotal for cholestasis diagnostic [24].

Flavonoids showed a good performance in hepatoprotection since almost all compounds showed a marked serum decrease of the liver and ductal enzymes (about 50% on average). In accordance with these data, it seems that genistein (5 μg/rat/day, p.o.) and quercetin (150 μmol/kg/day, i.p.) had a better performance than other flavonoids; however, intraperitoneal high dose (about 45 mg daily), and a confusing mode of administration (it began four weeks before biliary obstruction and was continued for a further four weeks) were inconclusive [25,26].

It is important to mention that quantification of metabolic biomarkers such as cholesterol, depicts indirect improvements on bile acid synthesis; however, metabolites such as glucose, triglycerides, and lipoproteins might determine a correct function of the liver, since this organ plays a central role in lipid and carbohydrate metabolism [24]. As seen above, few investigations consider metabolic parameters as pivotal endpoints for the evaluation of efficacy. Only three studies measured serum cholesterol, two measured triglycerides, and one measured lipoprotein content (LDL and HDL) (Table 3).

Further, it was remarkable to consider that risk of bias assessment show that the majority of studies assessed had a high risk for selection, performance, and detection biases (Figure S1). This evidence exposes an unmet need to improve the quality of the experimental protocols for most confident information in drug discovery. This was another reason for not including a meta-analysis in this investigation.

### 3.2. Experimental Study

#### 3.2.1. Signature of Obstructive Cholestasis in the Rat

**Figure 3:**
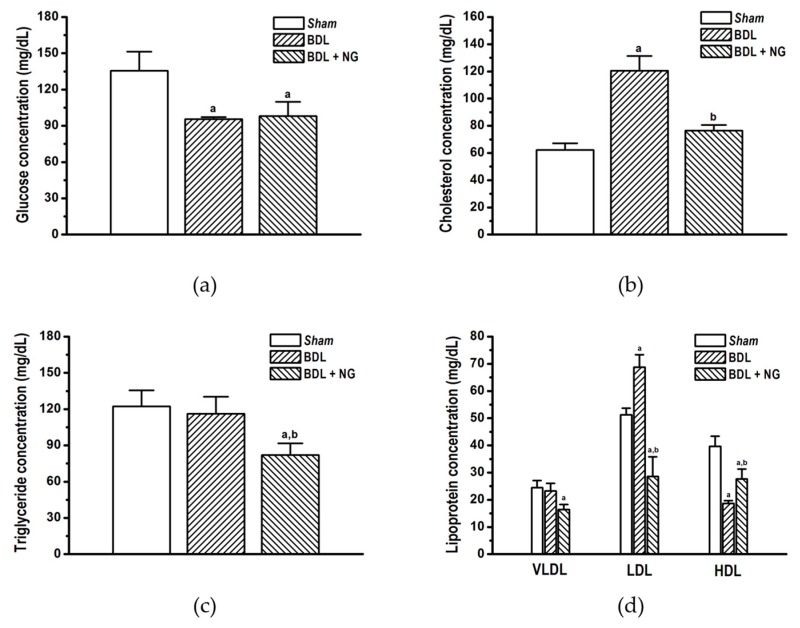
Metabolic biomarkers quantified on bile duct-ligated (BDL) model after administration of naringenin (NG, 50 mg/kg/day) for 10 days. (**a**) Glucose, (**b**) total cholesterol, (**c**) triglycerides, (**d**) lipoprotein (very low-density lipoprotein (VLDL), low-density lipoprotein (LDL), and high-density lipoprotein (HDL)). Data are expressed as means ± SEM (*n* = 8–10). a: Significantly different from sham control group at *p* < 0.05, and b: Significantly different from BDL group at *p* < 0.05.

**Figure 4:**
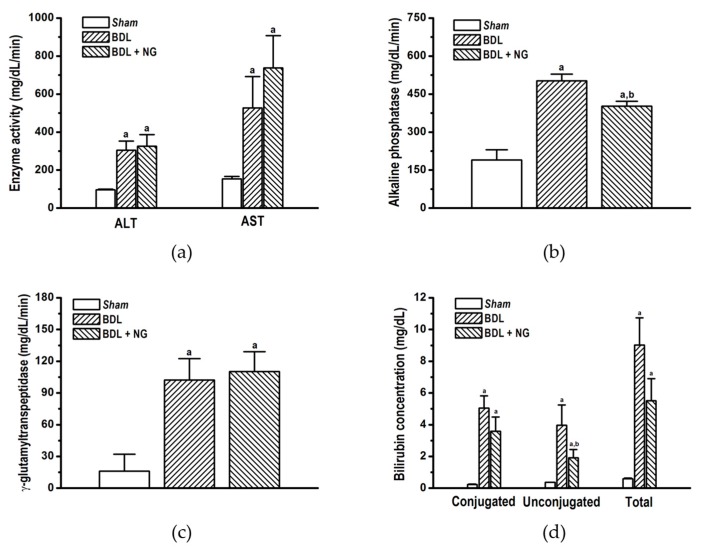
Liver function and cholestasis biomarkers quantified on bile duct-ligated (BDL) model after administration of naringenin (NG, 50 mg/kg/day) for 10 days. (**a**) Transaminases (ALT and AST), (**b**) alkaline phosphatase, (**c**) γ-glutamyltranspeptidase (**d**), and bilirubin were expressed from each group. Data are expressed as means ± SEM (*n* = 8–10). a: Significantly different from sham control group at *p* < 0.05, and b: Significantly different from BDL group at *p* < 0.05.

As reported, bile duct obstruction produced significant alterations in serum biomarkers concentration compared with sham-operated rats. Major alterations were decrease of glucose (Figure 3a) and HDL (Figure 3d), as well as increase of total cholesterol (Figure 3b), LDL (Figure 3d), and bilirrubin (Figure 4d) in comparison with the sham group. Triglycerides and VLDL concentrations were unaltered similar to the report by Stedman et al. [27]. Increase of liver enzymes ALT and AST, and bile ductal enzymes GGT and AP were determinant for cholestatic liver disease signature in BDL (Figure 4), due to hepatocellular damage produced by high concentrations of toxic bile acids [28].

#### 3.2.2. Efficacy of NG in Obstructive Cholestasis

Naringenin significantly improved serum cholesterol (Figure 4b) and low and high-density lipoproteins (Figure 4d) in the BDL model. Interestingly, despite triglycerides and VLDL were not modified on cholestatic damage, this flavonoid decreased both parameters. Additionally, significant decrease in bilirubin (Figure 4d) and AP activity (Figure 4b) suggests an improvement of liver and bile duct damage.

**Figure 5:**
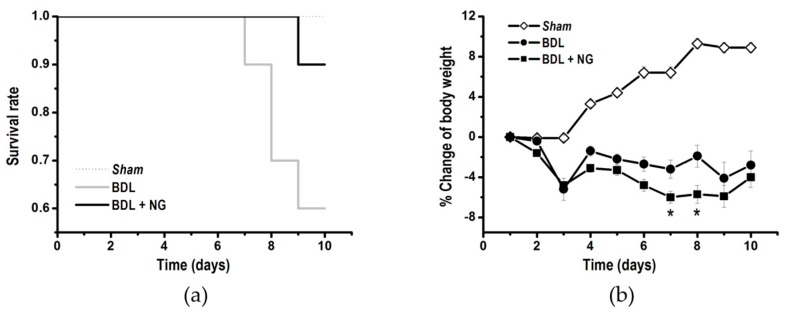
Survival rate (**a**), and percentage change of body weight (**b**) measured after administration of naringenin (NG, 50 mg/kg/day) for 10 days. *Significantly different from bile duct-ligated (BDL) group at *p* < 0.05 (*n* = 10 animals/group).

Finally, biochemical improvements attributed to NG allowed reduced mortality. By contrast, body weight changes along time had no modification. In fact, treated BDL rats suffered more weight loss than the BDL group (Figure 5).

## 4. Discussion

Flavonoids are naturally occurring compounds with beneficial health effects in chronic diseases, being cardiovascular and metabolic diseases that are better characterized. Further, higher intake of these compounds has been associated with lower mortality rates from specific vascular diseases and cancer [29]. The importance of flavonoid-rich foods consumption in preventing cholestasis mortality remains uncertain. For this reason, we aimed to show the state-of-the-art of flavonoids for the drug design of new anti-cholestatic agents through a systematic review.

Currently, some articles have applied systematic searching in order to analyze proficiency of natural products on specific illnesses (e.g. triterpenes on wound healing) [30]. Recently, Chen et al. reviewed phenylated flavonoids and their biological effects; this investigation focused on structural properties of the phenylated flavonoids and the implication in biological activity. However, no evidence was found on cholestatic liver disease or liver disease related abnormalities [13]. For this reason, the anti-cholestatic effect of flavonoids remains an outstanding issue in drug discovery.

Silymarin has been the most studied flavonoid for liver disease. This extract has been characterized as a potent antioxidant, immunomodulatory, and anti-fibrotic agent indicated as adjuvant treatment for all-causes liver disorders [11]. Our analysis shows that one major component of this extract, silibinin or silybin, improved serum transaminase, AP, and bilirubin in BDL model; which is possible by a decrease of pro-inflammatory lipids (platelet-activating factor, PAF), and improved activity and expression of both lysophosphatidylcholine acyltransferase LPCAT1 and LPCAT2, key enzymes in PAF remodeling [31]. Despite evidence, there is no more investigation about flavonoid-based therapeutic interventions for the treatment of cholestasis. This situation suggests an opportunity for medicinal chemists, computer-aided designers, or organic chemists to evaluate flavonoid as a potential scaffold for new lead compounds.

Our database search shows no evidence for evaluation of flavonoids efficacy and safety in a clinical setting. Only a pilot study (*n* = 27) was found where oral silymarin was assessed in UDCA non-responsive primary biliary cirrhosis (PBC) patients. Despite UDCA therapy for 52.6 ± 10.4 months, elevation of AP activity (2 times above normal) was persistent, neither adjuvant therapy with silymarin improved clinical endpoints [11]. In this context, diosmin, quercetin, or genistein should be good options since these flavonoids decreased AP activity 40–50% (Table 2). However, more clinical investigation is still needed to assess this potential.

On the other hand, eleven reports were retrieved from systematic search. The analysis showed a variety of administration and time framework, i.e., some reported pre-operative (one week) and post-operative (three weeks) administration [19,20]. This approach might bias in the therapeutic effect assessment, since it began one week before a cholestatic pattern is established. Additionally, animal species were different and possibly crucial for biological behavior. The most used is the Wistar rat (six studies) followed by Sprague Dawley (three studies). Despite Sprague Dawley was developed from Wistar strain, data show that Wistar strain is prone for metabolic impairments [32]. This should be relevant in taking into account the metabolic component of cholestasis [33,34].

As mentioned, metabolic impairment is a pivotal alteration on obstructive cholestasis. Alterations in glucose, cholesterol, LDL, and HDL were observed in the BDL group (Figure 4). Modification of cholesterol and lipoprotein on cholestasis by bile duct ligation surgery is known [35]; also, high concentration of plasma insulin [36,37] and low concentration of glucose [38] have been reported for this model. In contrast, other studies reported contradictory values suggesting a wide metabolic disparity between species, rodent strain, and time of damage exposure [38,39]. In addition, it has been established that intrahepatic cholestasis of pregnancy (ICP) and PBC are associated with impaired metabolic profile, including glucose intolerance and dyslipidemia [33,34]. Our analysis showed few data on metabolic parameters for BDL (Table 3).

This is the first time that NG is evaluated in a BDL model, despite that a simple review about beneficial effects of this flavonoid in ethanol- and carbon tetrachloride-induced liver diseases and its putative mechanisms was published with no evidence about obstructive cholestasis [40]. Mulvihill and colleagues reported similar effects in their studies using LDL receptor-null (LDL^−/−^) mice fed with a Western high caloric diet, which expressed metabolic impairments as dyslipidemia (increased VLDL and LDL), hyperinsulinemia, and weight gain vs. wild-type littermates. Treatment with NG 1% or 3% (wt/wt) improved triglyceride, total cholesterol, and lipoprotein concentrations after four weeks. In contrast, HDL was unaltered in animals with the Western diet, and NG did not modify this condition [41]. Our findings contribute for the beneficial metabolic effects induced by this flavonoid, taking into account that cholestasis have a distinct etiology and target organ than the diet-induced dyslipidemia.

Finally, the precise mechanism by which flavonoids exert their hepatoprotective effects is unclear yet. However, there is evidence suggesting that flavonoids might improve imbalance in oxidant/antioxidant status. For example, Kabirifar et al. reported that quercetin might improve the antioxidant capacity of the liver tissue by decreasing the activity of NADPH oxidase 1 (NOX1), a major intracellular producer of reactive oxygen species [42], and the expression of its activator Rac1. Further, an increase of the antioxidant enzymes catalase and glutathione superoxide dismutase (SOD) was reported [43]. Lin et al. showed similar evidences where an elevated mRNA expression of Mn-SOD, Cu/Zn-SOD, and catalase was observed. [19] On the other hand, it has been reported that flavonoids exert a regulation of inflammatory response and deposition of extracellular matrix by the decrease of mitogenic IL-1β and fibrogenic TGF-β1 expression and down-regulated BDL-induced hepatic Smad2/3 phosphorylation. [19] More investigation must be encouraged to promote comprehensive studies that describe the beneficial effects of flavonoids and its mechanism.

## 5. Conclusions

Flavonoids comprise a group of natural products with promising efficacy for the management of cholestatic liver disease. More clinical and experimental investigation should be driven to promote the drug design of flavonoid-based pharmaceuticals for the treatment of these pathologies with the lowest risk of bias. Naringenin is a flavonoid with specific beneficial effects on liver function and metabolic flow altered in cholestasis. Further investigation needs is required to characterize its pharmacological mechanism and applicability in a clinical setting.

## Figures and Tables

**Table 1:** Efficacy endpoints studied on selected articles of systematic review.

No	Chemical Structure	Compound Name	Dose Applied	Species	Efficacy Endpoints				Reference
					Liver Function	Fibrosis	Oxidative Stress	Inflammation	
1	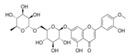	Diosmin	100 mg/kg/day, p.o. for 28 days	Wistar rat	X	X	X	X	Ali et al 2018
2	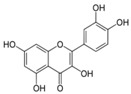	Quercetin	30 mg/kg/day p.o. for 28 days	Wistar rat	X	X	X		Kabirifar et al 2017
3			25 mg/kg/day p.o. for 28 days *	Sprague Dawley rat	X	X	X	X	Lin et al 2014
4			75, 150, 300 μmol/kg/day i.p. for 28 days	Wistar rat	X	X	X		Peres et al 2000
5	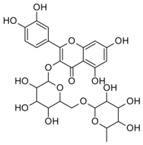	Rutin	25mg/kg/day p.o. for 28 days *	Sprague Dawley rat	X	X	X	X	Pan et al 2014
6	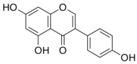	Genistein	5 μg/rat/day p.o. for 56 days	Wistar rat	X	X			Salas et al 2007
7	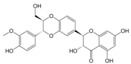	Silybin/Silibinin	0.4g/kg ad libitum p.o. for 28 days	Wistar rat			X		Serviddio et al 2014
8			Dose not determined for 28 days	Wistar rat				X	Stanca et al 2013
9	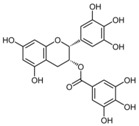	Epigallo- catechin 3-gallate	30mg/kg/day i.p. for 14 days **	C57BL/6 mice		X	X	X	Shen et al 2015
10			25 mg/kg/day p.o. for 14 days	Sprague Dawley rat	X	X		X	Yu et al 2015
11	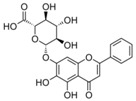	Baicalin	50 mg/kg/day i.p. for 14 days **	C57BL/6 mice		X	X	X	Shen et al 2017

* Compound administration began 1 week before surgery and 3 weeks postoperative. ** Compound administration began 2 h before surgery and 14 days postoperative.

**Table 2:** Percentage changes of liver function parameters induced by flavonoids on BDL model.

No	Reference	Compound	Change %					
			ALT	AST	AP	GGT	Total Bilirubin	Mortality
1	Ali et al 2018	Diosmin	48.77	48.32	50.53	46.37	56.77	20%
2	Kabirifar et al 2017	Quercetin	32.89	34.32	44.44	ND	ND	ND
3	Lin et al 2014	Quercetin	30.05	35.98	ND	55.87	36.26	ND
4	Peres et al 2000 *	Quercetin	55.81	78.57	49.86	ND	ND	ND
5	Pan et al 2014	Rutin	37.16	44.55	ND	63.49	40.66	ND
6	Salas et al 2007	Genistein	51.53	ND	59.88	73.68	68.97	ND
7	Serviddio et al 2014	Silybin/Silibinin	ND	ND	ND	ND	ND	ND
8	Stanca et al 2013	Silybin/Silibinin	30.76	23.68	27.78	ND	39.76	ND
9	Shen et al 2015	Epigallocatechin 3-Gallate	ND	ND	ND	ND	ND	ND
10	Yu et al 2015	Epigallocatechin 3-Gallate	−29.77	3.66	ND	ND	-4.64	ND
11	Shen et al 2017	Baicalin	ND	ND	ND	ND	ND	ND
12	Current article	NG	−6.79	−40.02	19.78	−7.82	38.91	30%

ND, not determined; NG, Naringenin; ALT, alanine transaminase; AST, aspartate transaminase; AP, alkaline phosphatase; GGT, gamma glutamyltranspeptidase. * Only was considered dose 150 µg/mL for this analysis since this dose was administered for 28 days. Negative values means an augmentation of the parameter in comparison with untreated cholestatic animals.

**Table 3:** Percentage changes of metabolic parameters induced by flavonoids on BDL model.

No	Reference	Compound	Change %						
			Glucose	Cholesterol	Triglycerides	Insulin	VLDL	LDL	HDL
3	Lin et al 2014	Quercetin	ND	14.78	26.47	ND	ND	ND	ND
5	Pan et al 2014	Rutin	ND	19.13	25.00	ND	ND	ND	ND
10	Yu et al 2015	Epigallocatechin 3-Gallate	ND	0.69	ND	ND	ND	0.74	−18.52
12	Current article	NG	−2.51	36.33	29.43	44.44	29.43	58.44	−48.50

ND, not determined; NG, naringenin; VLDL, very low density lipoprotein; LDL, low density lipoprotein; HDL, high density lipoprotein. Negative values means an augmentation of the parameter in comparison with untreated cholestatic animals.

## Supplementary Materials

The following are available online at https://www.mdpi.com/2218-273X/9/3/102/s1, Figure S1: SYRCLE’s tool for assessing risk of bias of the selected studies, Table S1: PICO strategy developed for systematic review.
