# Supplementary material for: Analysis of Flavonoids Bioactivity for Cholestatic Liver Disease: Systematic Literature Search and Experimental Approaches
Source: Biomolecules. 2019 Mar 14;9(3):102. doi: 10.3390/biom9030102 (PMC6468533; doi:10.3390/biom9030102)
Supplement: Supplementary file 1 [file biomolecules-09-00102-s001.pdf]

Supplementary Material

Table S1. PICO strategy developed for systematic review.

PICO term	Description
Patient	Human or animal with cholestasis or bile duct ligation
Intervention	Flavonoid*
Comparator	Flavonoid*, placebo or without comparator
Outcomes	Liver transaminases (ALT, AST), alkaline phosphatase (AP), γ-glutamyltranspeptidase (GGT), glucose, cholesterol, triglycerides, VLDL, LDL, HDL, mortality, and weight changes

*Flavonoid compound was defined as any of the following isomeric group: flavone, flavanone, isoflavanone, catechin, flavolignan, anthocyanin, benzoflavone, chalcone, flavonol, isoflavone, proanthocyanidin. Any isomeric compound was considered for this review.

Figure S1. SYRCLE's tool for assessing risk of bias of the selected studies.

	Sequence generation (selection bias)	Baseline characteristics (selection bias)	Allocation concealment (selection bias)	Random housing (performance bias)	Blinding (performance bias)	Random outcome assessment (detection bias)	Blinding (detection bias)	Incomplete outcome data (attrition bias)	Selective outcome reporting (reporting bias)*	Other sources of bias (other) †
Ali et al 2018	?	+	+	+	+	+	+	+	-	-
Kabirifar et al 2017	?	+	+	+	+	+	+	+	-	-
Lin et al 2014	?	+	+	+	+	+	+	+	-	+
Pan et al 2014	?	+	+	+	+	+	+	+	-	+
Peres et al 2000	+	+	+	+	+	+	+	+	-	-
Salas et al 2007	+	+	+	+	+	+	+	+	-	-
Serviddio et al 2014	+	+	+	+	+	+	+	-	-	-
Shen et al 2015	+	+	+	+	+	+	+	-	-	+
Shen et al 2017	+	+	+	+	+	+	?	-	-	+
Stanca et al 2013	+	+	+	+	+	+	+	?	?	-
Yu et al 2015	?	+	+	+	+	?	-	-	-	-

* All studies marked as low risk (minus sign) are denoted as "possibly yes" in accordance with response options suggested in Cochrane's RoB tool. † Studies indicated as high risk (plus sign) reported an inconsistent mode of administration of the sample as described in Table 1.
